# Accountability and objectivity: Humanitarian narratives at the intersection of evidence and localisation

**DOI:** 10.1186/s41018-024-00160-x

**Published:** 2024-12-28

**Authors:** Ellen Turner, Michelle Lokot, Isabelle L. Lange, Caitlin Wake, Bayard Roberts

**Affiliations:** 1 Faculty of Public Health and Policy, London School of Hygiene and Tropical Medicine, London, UK; 2 National Institute of Teaching, Blackburn, UK; 3 Department of Neurology, TUM University Hospital and Center for Global Health, TUM School of Medicine and Health, Technical University of Munich, Munich, Germany; 4 Humanitarian Policy Group, Overseas Development Institute, London, UK

**Keywords:** Use of evidence, Localisation, Humanitarian decision-making

## Abstract

In the last decade, there has been a push for greater evidence-based practice within the humanitarian sector, alongside an increasing turn towards localising humanitarian assistance. Humanitarian actors and organisations have been increasing their production and use of evidence, while also being encouraged to reflect more critically on power hierarchies and decolonise humanitarian aid. This paper explores the intersection of these two narratives, examining how the use of evidence in humanitarian decision-making fits within a localisation agenda. Based on interviews with humanitarian health practitioners located globally, we examine how evidence is defined, and how it is used, including to inform both hierarchical and bottom-up approaches to decision-making. We find clear hierarchies about what counts as good evidence, with a weighting towards randomised-controlled trials, and that the perspectives of populations most affected by crises and the expertise of local actors were not routinely seen as central forms of evidence. Narratives about needing to build the capacity of local actors persist, alongside the notion of evidence as objective. We suggest that a disconnect exists between humanitarian discourses about evidence and localisation, arguing for the need to view evidence as political and influenced by researcher positionality This suggests that more consideration of locally-driven knowledge is needed and will strengthen humanitarian decision-making. We argue that a distinction between evidence and localisation does a disservice to both agendas and that finding synergies between these concepts would strengthen both.

## Introduction

### A turn towards evidence in the humanitarian sector

Humanitarian assistance requires stakeholders to make a huge number of decisions, with very high stakes, in pressured situations and often with limited or patchy information to support decision-making. Decisions often need to be made about how to coordinate effectively, prioritise competing needs, maximise limited funds, ensure the safety of humanitarian actors, and manage complex dynamics and politics in challenging settings. The processes by which humanitarian actors make decisions, and the kinds of information they rely on to support these decisions, are rarely documented systematically or in detail. Evidence suggests that decision-making varies by individual, by context, and requires different types of thinking (Clarke and Campbell 2018; Comes 2016). A lack of clear decision-making structures risks that humanitarian decisions become ‘spontaneous, unstructured, and reactive’ (Comes 2016, p. 2). Over the past decade, there has been a push for greater evidence-based practice and decision-making within the humanitarian sector, mirroring a similar trend in other areas of public policy (Nutley et al. 2007; Stern et al. 2012). This has involved a focus on generating and using quality evidence to support decision-making (Ager et al. 2014; Blanchet et al. 2017; Dijkzeul et al. 2013), and international agencies and national funders laying out guidelines and strategies for increasing evidence-based practice in the sector (DFID 2014; UNFPA 2010). Organisations have shared guidance on how evidence can be used in the humanitarian sector (Blanchet et al. 2018).

In contrast to some other areas of public policy, however, within the humanitarian sector the focus on evidence has involved emphasis on its role in contributing to improved transparency and ‘upwards’ accountability to donors (Chynoweth 2015)*.* Following dramatic increases in humanitarian aid between 1990 and 2000, donors focused more closely on the coordination and performance of humanitarian action, and required increased regulation and monitoring of organisations receiving their aid (Macrae et al. 2002). Scholars suggest accountability became ‘skewed’ towards donors instead of towards populations receiving aid (Harrell-Bond 2002, p. 53). The reliance on results-based management tools, such as logframes, risked perpetuating simplistic, linear solutions to complex problems. As such, evidence has risked coming to be understood according to a narrow meaning of ‘what works’, without sufficient consideration of power dynamics, context and politics (Eyben 2013, p. 17). In humanitarian narratives, evidence is increasingly framed narrowly (including by donors), tied to concepts of causality, attribution, efficiency, value for money and rigour (Eyben 2013). Such evidence is often demonstrated quantitatively (Barnett 2011), with certain forms of knowledge being privileged over others (Lokot 2021).

Further, within humanitarian narratives about evidence, distinctions are often made between decision-making that is based on careful consideration of the evidence on the one hand, and intuitive, reactive decision-making, based on personal experience and conviction, on the other (Knox-Clarke and Darcy 2014; Knox Clarke and Campbell 2020). However, the evidence itself may be a product of data bias, as increasingly donors shape programmatic and research priorities, determining what issues warrant evidence generation, leaving other topics underfunded (Paulus et al. 2023). Paulus and colleagues (2023) document how competitiveness between humanitarian actors, pressures to make swift decisions and gaps in data can result in data being collected in biased ways, for example, designing sampling to prove an argument. Such evidence is itself political, yet is often problematically presented as objective and rational (Colombo and Checchi 2018; Knox Clarke and Campbell 2020). As Comes (2016) argues, humanitarian decision-making is beset by significant biases, including cognitive biases that result in over-simplification of complex problems, and confirmation biases that rely on information to affirm old hypotheses, meaning that how evidence is *used* is also political. Humanitarian decisions often occur by group consensus, creating further problems for how groups assess and verify evidence (Comes 2016; Knox-Clarke 2014). With these challenges around how evidence is generated and used, the very concept of ‘hard evidence’ comes into question (Eyben, 2011, p. 1), creating blurred lines between decision-making based on such evidence and decision-making based on other factors such as intuition and experience.

### Localisation and bottom-up approaches to decision-making

In the last 10–15 years, alongside the drive for evidence-based decision-making, there has been an increasing turn towards what is referred to as ‘localisation’ in humanitarian decision-making (Roepstorff 2020). Localisation has varied definitions. In this paper we use this often-used description, which sees localisation as: ‘a process of recognising, respecting and strengthening the leadership by local authorities and the capacity of local civil society in humanitarian action, in order to better address the needs of affected populations and to prepare national actors for future humanitarian responses’ (Fabbre 2017). The 2016 humanitarian ‘Grand Bargain’ formalised localisation, and saw representatives of 18 donor countries and 16 international aid organisations commit to channelling 25% of global humanitarian funding to local and national responders by 2020, and to work towards a ‘participation revolution’ where people receiving aid lead the decision-making that affects them. Participation of refugees is becoming an ‘emerging norm’ in the humanitarian sector (Milner et al. 2022, p. 567).

However, despite efforts to increase localisation, including shifting funding to national and local actors, progress in financially supporting localisation has been limited. For example, it was estimated that just 0.1% of humanitarian funding to respond to Covid-19 had gone to local and national actors (Charter4Change 2020). The 2022 State of the Humanitarian System report by the Active Learning Network for Accountability and Performance [ALNAP] found that only 36% of the recipients of humanitarian aid they surveyed felt that aid went to those who needed it most, with poor engagement with communities cited as contributing to mistrust and perceptions of exclusion (ALNAP 2022). Qualitative evidence from a range of humanitarian contexts has shown that progress has been slow and that the processes of localisation can continue to both exclude and disempower local actors and organisations (Anderson et al. 2012; Clark-Kazak 2010; Pincock et al. 2020; Usen 2019; Wilkinson et al. 2022), and even perpetuate imbalances of power through forms of instrumentalisation (Dixon et al. 2016; Fiddian-Qasmiyeh 2018), and surveillance of local actors (Mulder 2023). For example, in one study with a range of humanitarian actors working in the Syrian crisis, local organisations were generally viewed as a ‘risk to be managed’ by international actors and were handed the risks and challenges of making projects work, without any real agency (Dixon et al. 2016). In another study in Ethiopia, local actors were asked to show that as part of the project’s explicit localisation approach, their solutions were locally-driven by communities, despite having to work within hierarchical project structures that were driven by strict surveillance (Mulder 2023).

Relationships between international (often driven by funding from Western governments) and local and/or national organisations, are rooted in historical and inherent imbalances of power (De Torrenté, 2013; Kothari 2005; Taela 2023), that continue to shape the overall framework for the processes of localisation. This can be exacerbated by the often highly stressful and time-limited conditions in which humanitarian actors make decisions, and that can at times lead to feelings of defensiveness (Walkup 1997), and a reliance on personal intuition (Comes 2016; Knox Clarke and Campbell 2020). Further, in a globalised context where what constitutes knowledge and expertise is often determined by international actors and organisations (Sou, 2022; Mosse 2007, 2011), local and national actors may find that in the process of localisation, their expertise and capacities are assessed, somewhat paradoxically, according to criteria they did not set, and far from the context of the crisis (Fast and Bennett 2020; Fiddian-Qasmiyeh 2018). Some have also critiqued the term ‘local’, highlighting that it enforces a false dichotomy between local and global approaches, fails to recognise the heterogeneity of the wide range of stakeholders subsumed under the term ‘local’ (Robillard et al. 2020), and thus reinforces colonial thought underpinning imbalances of power (Roepstorff 2020).

There are challenges too to the meaningful participation of communities in decision-making, such as determining who is entitled to speak for a community, and who this representation may exclude (De Torrenté, 2013; Lokot 2021). Humanitarian actors have at times been criticised for poor attention to power dynamics within communities when trying to promote participation, linked to paternalistic assumptions about who holds power and the assumption that local actors are a homogenous group (Oliveira and Vearey 2020; Pincock and Bakunzi 2021). Efforts to include communities affected by crises in decision-making may also not fully account for the time and financial burden of participating. Increasingly, critiques of tokenistic participation as well as reflection on the real challenges and unintended consequences of involving refugees in decision-making, suggest that more work needs to be done to think through the practical implications of participation (Lokot et al. 2023).

Despite these challenges and shortcomings, centering the priorities of populations affected by crises, and the expertise of local actors, is shown to be central and key to humanitarian action. Reviews in health systems in humanitarian settings have found that responses are strengthened through bottom-up and community-based approaches (Durrance-Bagale et al. 2020; Lokot et al. 2022), with a systematic review finding that dominance of donor influence, an example of a top-down mechanism, was a key barrier to health systems governance (Lokot et al. 2022). Failings in humanitarian responses can be linked to poor understanding of sociocultural contexts in particular settings, and mistrust between international humanitarian actors and local populations (Colombo and Pavignani 2017).

We currently know little, however, about how the priorities of populations most affected by crises are reflected in humanitarian decision-making, both in terms of how this is valued by decision-makers, and the processes by which such evidence is gathered.

## The role of evidence in top-down and bottom-up approaches to decision-making

We also know little about intersections between the processes of localisation and evidence-based practice have intersected. In particular, how far the priorities of populations most affected by crises, and the expertise of local actors in responding to particular crises, are reflected in the kinds of evidence humanitarian actors use to make decisions. These questions involve examining what processes are used to gather insights into the views and experiences of populations affected by crises and local actors; how these forms of evidence are considered and valued by humanitarian decision-makers; and how they are included in the process of making key decisions. Further, a greater reliance on evidence in decision-making requires attendance to what constitutes evidence, how it is valued and whose voices are included. Decolonial critiques in the humanitarian and international development sectors are increasingly unpacking how research itself is imbued with deeply embedded imbalances of power and representation, historically shaped in colonial relations (Peace Direct 2021).

So far there has been little interrogation of the ways in which the push for use of evidence and for greater localisation in the humanitarian sector have intersected. While an increasing focus on evidence has the potential to reinforce top-down decision-making processes and structures, for example through setting a (top-down) standard of what ‘good practice’ is (Kothari 2005; Mosse 2007; Sou, 2022; Taela 2023), it may also have the potential to strengthen accountability to populations most affected by crises, for example through taking seriously the task of gathering and documenting their priorities.

Decolonial critiques in the humanitarian and international development sectors have shown how research may be deeply rooted in colonial structures and imbalances of power, and as such can be instrumental in reinforcing inequalities. Rooted in a long legacy of research and representation being key to the colonial project (Said 1978), research may be a key way in which stereotypical notions are reinforced (Lokot 2019). A focus on using evidence to support practice is concerned with elucidating good, effective practice, and involves identifying those who are permitted to speak with authority (Mosse 2007). Mosse explores how international development discourses produce particular kinds of global knowledge, ‘experts’ that speak on this knowledge, and patrol the boundaries of this knowledge and how others are ranked against it (Mosse 2007, 2011). While ‘local’ forms of knowledge are portrayed as value-laden and context-specific, expert global knowledge, such as can be seen in international agency frameworks and guidelines, are viewed as transcending norms and values and are used authoritatively across settings. Greater professionalisation and regulation of the humanitarian sector has been argued to lead to a depoliticisation of the international development project, concealing the implicit reinforcement of the authority of Western governments and agencies (Mosse 2007), and a ‘tidying up’ of the messiness of lived experience (Kothari 2005). Kothari (2005) examines how a focus on ‘expert knowledge’, rooted in colonialism, mutually reinforces the legitimacy of the actor, and of the action, and so doing, the neoliberal development agenda. What counts as evidence, and how it is operationalised, cannot, therefore, be easily extricated from broader imbalances of power and colonial legacies.

Further, the process of research itself may also further reinscribe such inequities. Research can lead to exploitation or marginalisation of researchers working in contexts of humanitarian crises, through research agendas and funding allocation that are set far from the setting of research itself—often in institutions in the Global North—and that offer researchers little agency or meaningful engagement in the research design and dissemination (Sukarieh and Tannock 2019). Lack of meaningful involvement with all stakeholders in the research process can lead to certain forms of knowledge being valued over others, top-down decision-making, and extractive relationships with communities close to the research (Lokot and Wake 2021). Such inequities, while not unique to humanitarian research, may nevertheless be exacerbated by the urgency and complexity of humanitarian crises (Lokot and Wake 2021; Sibai et al. 2019). The question of what constitutes good evidence, what it says about humanitarian action, and who is allowed to say it, is therefore far from simple in its relationship to the localisation agenda.

However, emerging research is beginning to show how methods can be used to elucidate the priorities of populations most affected by crises, and prioritise the meaningful involvement of all stakeholders in the research process itself. Greater efforts are being taken to focus on practical ways in which the hierarchies and imbalances of power in research can be addressed, and how they can be used to serve communities (Lokot and Wake 2021). Researchers are increasingly attending to decolonial critiques of methods used to generate evidence, drawing on indigenous forms of knowledge to generate evidence in humanitarian settings (Cuaton and Su 2020; Hoffman 2021; Khumalo and Munsaka 2021). There is also more recognition that reflexivity in the humanitarian sector is an important part of unravelling assumptions about research objectivity (Lokot 2022). The overall aim of the paper is to explore the use of evidence in humanitarian decision-making within the context of a localisation agenda. The objectives are to explore the kinds of ‘evidence’ humanitarian actors rely upon, how they conceptualise evidence, and to identify if and how the priorities of populations most affected by crises and the expertise of local actors are reflected in humanitarian decision-making.

## Methodology

This study examines interviews with 14 international humanitarian actors to identify the kinds of evidence that international humanitarian actors draw on when making decisions, and how the priorities of populations most affected by crises, as well as the expertise of local actors, are reflected in humanitarian decision-making.

We build on research conducted within the RECAP [Research capacity strengthening and knowledge generation to support preparedness and response to humanitarian crises and epidemics] research study (London School of Hygiene and Tropical Medicine, n.d.). RECAP was a collaboration between the London School of Hygiene and Tropical Medicine, the American University in Beirut, the University of Sierra Leone, and a network of further universities and humanitarian non-governmental organisations [NGOs], with the overarching aim of improving how data are used in humanitarian settings, and the use of co-production approaches to address power hierarchies in humanitarian decision-making (https://www.lshtm.ac.uk/research/centres-projects-groups/recap). The interviews we draw on in this analysis were initially conducted by IL to examine how evidence is defined and valued by humanitarian practitioners, how this has evolved over time, to identify areas of innovation. Due to the rich insights of the data generated through the initial analysis, we saw opportunities to examine in further depth how humanitarian actors’ use of evidence intersected with a concern with making humanitarian decision-making representative of the views and priorities of populations most affected by crises, and local experts. We thus conducted further analysis to delve deeper into these areas. The specific research questions guiding this analysis are:

1. To identify the kinds of evidence humanitarian actors rely upon to make evidence-based decisions during crises;
2. To understand how the use of evidence fits within a localisation agenda;
3. To explore how the priorities of populations most affected by crises, as well as the expertise of local actors, are reflected in humanitarian decision-making.

In total, 14 participants were interviewed by IL and CW in 2020–2021 in English. Participants were purposively sampled for their involvement in the design, implementation and monitoring of health interventions in humanitarian crises, and for their long-term experience of working within the humanitarian sector. Long-term and diverse experience in the humanitarian sector was essential for the research, as reflecting on the use of evidence in the sector over time was a key aspect of the original research aims. Participants were identified through networks of senior members of the RECAP study team, and approached by members of the research team via email. Participants were located in geographically diverse locations and had a range of experience working in the sector, with diverse roles spanning academia and humanitarian practice and at times both. At the time of the research, participants were employed at universities (*N* = 3), international NGOs [INGOs] working in humanitarian practice (*N* = 6), international agencies (*N* = 3), and international humanitarian research organisations (*N* = 2). Nine participants were female and five participants were male, and most had over 20 years of experience working in the sector.

Interviews were semi-structured in nature, to allow a focus on the key areas of interest to the research, while also allowing participants to reflect on topics and perspectives that were important to them and pertinent to their experience. As such, interview topic guides included questions on what counts as evidence, how it is defined and whether that has changed over time; how participants use evidence in their practice, including dilemmas and challenges; how evidence features in the processes of localisation; and flows of evidence and accountability; however, they were also structured to encourage and respond to participants’ own narratives. Interviews were conducted over the phone, face-to-face, and over an online platform, such as Zoom, with an opportunity for follow-up questions or comments by email or over the phone. Interviews were conducted in English and digitally recorded and transcribed with participants’ consent, with quality checks for transcription. The study received ethical approval from the Ethics Committee of the London School of Hygiene and Tropical Medicine.

The analysis was conducted in several stages and was iterative in nature. A first round of analysis was conducted by IL and CW in 2021 and examined the use of evidence over time in the humanitarian sector. A second process of analysis explored several key themes in further depth in 2023, and formed the basis of this paper. This analysis process included several stages. First, ML and BR determined key areas of interest for analysis based on the first round of analysis; second, ML and ET read across the transcripts and generated an initial list of overarching codes, meeting research objectives; third ET, in collaboration with ML, inductively coded the transcripts drawing on various grounded theory approaches as synthesised by Eaves (2001). This included line-by-line in vivo coding using participants’ own language, using constant comparison to identify relationships between codes, and developing categories developed into ‘mini-theories’ based on the codes. ET and ML collaboratively reflected on and synthesised the ‘top-down’ approach to generating codes based on the initial read of the transcripts and areas of interest to the research questions, and the ‘bottom-up’ in vivo coding using participants’ own language, at several stages in the process, generating a final code list. The final code list was shared with BR and IL for their interpretive input. Overall, interviews were complex and heterogeneous, as participants reflected on a wide range of experiences in the sector, and shared diverse, at times contradictory views. Some spoke personally, and others drew more on more institutional perspectives of the organisations they worked for. This heterogeneity was sought out and explored in the analysis, and contradictions and complexity were examined at each stage.

Our study was limited by several factors. Firstly, we draw on a relatively small sample of 14 interviews of practitioners within a narrow field (and none from local humanitarian organisations), however, the participants contributed rich insights and represent a wealth of varied experience spanning several decades. Secondly, the data was collected during acute phases of the COVID-19 pandemic, leading to some actors initially being unavailable for participation, and data collection being spread out over many months to accommodate their participation. Thirdly, these interviews were conducted in 2020-2021. While the issues raised appear very topical and reflect in many cases deep-rooted, systemic issues within the humanitarian sector, it is possible that more recent developments may be missed in the data. Lastly, while some of our authorship team have experience as humanitarian practitioners, our study team is based in an academic institution, therefore the lens we use may be different from those who are currently working in humanitarian practice. 

## Findings

### Framing and defining evidence

The participants discussed what they considered to be evidence in a number of ways. First, they described clear hierarchies between different forms of evidence. Most participants agreed that humanitarian actors’ personal intuition and previous experience did not count as evidence:

‘[E]vidence is something that is repeatable, obviously done in different contexts and shows effectiveness for something. That’s the cornerstone of it. It’s something that has been done in many places ideally and it’s not just one person’s opinion’ (female participant, INGO, 2).

‘[Evidence is] not only based on personal experience, I think that would be the simplest way to say it. That it’s not just based on personal experience, or on let’s say, tradition that we are used to custom. […] Those are the two things that would make evidence different from the other information’ (male participant, international agency, 6).

Others, however, viewed personal intuition as an important aspect of decision-making, but lower on the hierarchy of evidence than other forms of evidence:

‘…your gut feeling, though, your personal experience. I think that is definitely that is definitely evidence, just that it perhaps at a low… it’s somewhere lower in the hierarchy of evidence. And it’s really subject to cognitive biases of various types but I definitely use it myself’ (male participant, academia, 10).

On the whole, participants mentioned impact evaluation research into the effectiveness of different interventions as the most rigorous form of evidence, underpinned by concepts of scientific ‘truth’. This was generally viewed by participants as the highest quality evidence, and the term ‘evidence-based’ was often used as a shorthand for this form of research:

‘What I would say is the operational definition of evidence. That’s quite broad. That means that we have credible information that what we do, the intervention, has the effect or is likely to have the effect that we intend. That’s the kind of evidence that I use’ [male participant, international agency, 6].

‘I think that most people would have that kind of knee jerk response, and evidence [is] some kind of scientific research that that shows you something is, sort of true and valid [that has] come through experimentation and research, or something like that – is proven true’ (male participant, INGO, 12).

As seen here, references to ‘most people’, point to a general acknowledgment that this was how evidence was viewed in the sector, suggesting discursive relevance to these notions of evidence. Indeed, several participants positioned themselves outside this view of evidence that they saw as widespread, but narrow:

‘[W]e don’t subscribe to the view that evidence is only that which is produced through an impact study or a quasi-control or experimental design which is how some people would define evidence. The reason why we don’t define that is because […] it depends on the question’ (female participant, research organisation, 3).

‘I prefer seeing a lot of evidence, some of which is not from RCTs [randomised-controlled trials] and kind of grading the evidence and using basically using all of the evidence there is […]. So it’s not evidence on interventions, which interventions work […] it’s more what do you do with the public health information that you have […] A lot of [evidence] is very focused on […] research on interventions, which I think is really the tip of the iceberg […] But I don’t think many people take that broader view of evidence’ (male participant, academia, 10).

These participants both construct here a notion of what is largely considered evidence in the sector, with a weighting towards RCTs and impact evaluations, and position themselves as considering broader forms of evidence. While the former participant focuses on the decision itself and the suitability of different forms of evidence, the latter describes drawing on public health information to understand the context. Such narratives could be seen to both show the predominance of discourses on hierarchies of evidence and also point to how they were not fixed.

There was also an acknowledgement that RCTs as a form of evidence required a level of rigour and specificity that was not always suited to humanitarian research contexts and questions. Some participants felt that an RCT design excluded key populations, or were not able to answer important questions.

Other forms of formalised evidence, such as qualitative data, were also discussed, but less often. Further, qualitative evidence did not tend to be given as examples of ‘evidence-based’ approaches, and was described as being less influential. For example, one participant suggested when comparing qualitative versus quantitative research, ‘quantitative weighs more’ (female participant, INGO, 1). It was interesting that qualitative research was discussed less often in conversations around evidence, while research suggests that qualitative research has tended to be the most commonly used method in humanitarian evaluation research (Knox Clarke and Darcy, 2014).

Routinely collected health data was also discussed by some participants; however, this was described at times to be challenging to access or inaccurate: ‘[I]t’s not that I didn’t have access to the data, it’s that the data was completely wrong in some places’ (female participant, INGO 13). Some participants also discussed how conducting needs assessments enabled them to determine actions, while others suggested needs assessments were just a starting point and needed to be supplemented with other data:

‘[N]eeds assessment is one piece of the many other pieces one needs to consider. Needs assessments may say that people are not accessing healthcare even when they are sick or going to whatever locally available but that doesn’t necessarily tell us what intervention will be effective’ (male participant, INGO, 9).

Another key form of evidence that participants discussed was international guidelines, synthesising and drawing on prior research. This was often viewed as being expert knowledge that was supported by research into programme effectiveness:

‘All our strategies are based on evidence and those are either interagency or WHO [World Health Organisation] […] Everything that we’ve put together has been evidence-based and constantly scouring WHO and [other] guidance […] It’s kept changing, so we’ve had to continually ensure that we are changing our guidance according to the newest thinking and best practice that is available. Sometimes it’s not evidence-based and it’s the best that we know of at the time. I guess, we would say expert evidence’ (female participant, INGO, 2).

In describing what counts as evidence, international guidelines are often discussed, as shown here, as a useful touchpoint for ‘newest thinking’ and the ‘best’ and most ‘expert' evidence that is available. The mention of ‘sometimes it’s not evidence-based and it’s the best we know’, points to the specificity of how this was considered, and the hierarchies this involved.

Most participants did not explicitly mention including the views of populations receiving humanitarian assistance or the expertise of local actors to be key forms of evidence. A small number did, however, and felt the need for this strongly:

‘Best evidence is […] good scientific studies complemented with clinical expertise, with my empirical experience and analytical thinking, and so on and so forth. But also I think that evidence… this is the best evidence for [country name]. This evidence should be also involving my clients’ views- what are accessible, what are acceptable, what are culturally relevant to them? So I think this is the evidence with the three main components’ (female participant, academia, 11).

Participants, therefore, on the whole, described a general, internationally recognised standard for what constituted high-quality evidence, and that using evidence to make decisions in humanitarian contexts could not always meet these standards. There was clear discursive relevance for ‘evidence-based’, referring to certain forms of research, predominantly impact evaluations and RCTs, while others, such as qualitative, needs assessments or routinely-collected data, and previous experience or intuition, were considered lower down the hierarchies of evidence. Evidence focusing on the priorities and perspectives of populations most affected by crises, or the views and expertise of local actors, were not often explicitly mentioned as forms of evidence, and did not tend to feature in conversations about ‘evidence-based’ approaches.

### Use of evidence in decision-making

In spite of the discursive relevance discussed above for the term ‘evidence-based’, and its association with particular forms of formalised research, participants described synthesising and drawing on a range of evidence sources when making decisions. These included formalised evidence about interventions that have been found to be effective elsewhere; information or knowledge about the context; and decision-makers’ own expertise or intuition. For some, this mix was viewed in quite formalised terms:

‘I would say it’s fairly balanced to one-third distribution of what have we done on a particular type of problem before, drawing on previous program design. [O]ne-third would be needs assessment or any new information that we can collect, hands-on, and then one-third would be the narratives to fill the gaps’ (male participant, INGO, 9).

In this example, narratives were viewed to be beliefs based on prior experience or understanding of the concept, that was not necessarily tied to specific evidence. For others, however, this mix was more informalised and less clearly demarcated:

‘I think a lot of things [are] considered evidence and I think it’s extraordinarily organisationally-specific and person-specific and context-specific’ (female participant, research organisation, 8).

‘It’s my thought process. I’m in the middle of it. It’s just a lot of things are still in the process of being thought through and some of it has a link with my experience, and then others have a link with the research. It’s a mix, but it’s always a mix even when you’re practitioning [*sic*] I think. The mix [has] different proportions, but it’s still a mix’ (female participant, INGO, 13).

International guidelines were often described to be drawn on as the backdrop for decision-making, that was then synthesised, adapted and considered in light of other forms of information and expertise.

Several participants described the importance of adapting to context. While still relying on RCTs as shorthand for the highest quality evidence, there was the view that such evidence needed to be interpreted in particular contexts:

‘It’s not just that you see in one or two randomised control trials about an intervention that works and then you say, “Okay, then I must do it.” You need to be convinced that it makes sense in the setting that you are doing it, you need to believe in it, so to say, and that is based on sometimes factors outside the formal evidence base’ (male participant, international agency, 6).

Interestingly, as this participant describes here, while RCTs were held to be the highest quality form of evidence, there was also a general view that other ‘factors’ were also crucial in order to operationalise this formalised research and evidence in decision-making. Across the data, participants tended to position such ‘factors’, usually referring to information about the context, and previous experience or intuition, as not evidence, or ‘outside the formal evidence base’, but nonetheless crucial in making formal evidence useful in decision-making.

At times, participants also described that it was essential to understand local contexts of research as this had a bearing on how activities were implemented. This tended to be more often discussed in terms of the context of interventions, however, and information about local context was not necessarily seen as a form of evidence. In the following example, a participant working with an INGO context described how programming needed to be shaped by crucial information about the local context:

‘Politics definitely influences the decision but how evidence can fit in there is probably less thought of […] Let’s say Uganda is now hosting so many refugees so the local tensions are between the host and refugee communities and are taken into account. That the new resources coming in for humanitarian programming should allocate more services for both refugee and host communities and not be biased against host communities at equity angle. I don’t think evidence also plays role there. There’s different types of evidence but people recognise that there were local tensions that’s hampering service delivery’ (male participant, INGO, 9).

Interestingly, despite insights into dynamics between refugee and host communities that are described here as essential for appropriate programming, such insights are not described as evidence, and indeed positioned as the opposite of evidence (‘I don’t think evidence also plays a role there’). Often in these discussions, it was unclear how such information was gathered, and how it was documented and travelled to decision-makers. Despite knowledge of the context being described as essential to appropriate decision-making, it was therefore not generally considered to be evidence and little specificity was given as to how this evidence was generated and used, or who it involved.

Overall, therefore, participants described drawing on a range, or a ‘mix’ of forms of evidence to make decisions; however, there were clear hierarchies in how these forms of evidence were viewed. There was a general sense among participants that making decisions based on such forms of evidence required a certain level of juggling and pragmatism, and that the ‘ideal’ research was not always possible:

‘It’s not going to be a definitive approach. Evidence-based can come proactively through field data collection or retrospectively, and it’s just, “Okay, can we design better tools? […] Is not maybe as solid like a randomised clinical trial, but it’s the best we can do with the resources we have and given the context’ (female participant, INGO, 14).

‘[T]hat level of whether uncertainty or absence of evidence, or at least relative absence, or lack of evidence, or conflicting evidence, is something that we deal with, and we need to make decisions and choice. Not necessarily, entirely informed, but this is the best we can have at the moment’ (male participant, international agency, 5).

### The role of evidence within top-down approaches to decision-making

Approaches to decision-making within the humanitarian sector were described as highly top-down in nature, driven by donor interests, priorities and funding cycles, and by international agencies. In participant accounts, at some points those at the top were donors and at other times, they were international agencies; at some points, those at the bottom were described as local responders and at other times they were communities—suggesting multiple layers of hierarchies. While some donors were described to be more flexible than others, across the data there was widespread acknowledgement and discussion of the ways in which funding shaped decision-making from the top down:

‘It depends on the donor but some donors are really really not flexible, they have a clear vision and they want that you to implement their vision’ (female participant, academia+, 11).

‘That way of funding humanitarian responses, the factor also really constrains decision making and use of evidence because the process is very top-down, you are given a budget, an allocation, you are told to work in a certain location and you have to fit your project within those boundaries, you have to’ (male participant, academia+, 10).

On the whole, participants were highly critical of top-down decision-making structures, and there was widespread agreement that the sector oriented towards accountability to donor organisations, rather than communities receiving humanitarian assistance. Despite a general and widespread critique of this approach, participants varied widely in how they viewed the role of evidence in this. Evidence was viewed by participants as central within this top-down dynamic of decision-making, however the role it was viewed to play in relation to top-down decision-making was multifaceted and at times contradictory.

Despite the fact that across the data, participants were broadly critical of top-down, donor-driven approaches, some participants felt that increased focus on professionalisation in the humanitarian sector and increasing top-down accountability was a necessary and important step in strengthening the sector. Here, evidence was portrayed as a way in which poor practice could be counteracted, from the top down:

‘There’s so many examples I think where the social norms I was talking about actually end up rearing their ugly head and working in the counter in the other direction. People who aren’t literate enough and savvy enough with this stuff, they just cling onto whatever number is given them […] I think that evidence is always going to be competing with or have to contend with the very strong biases and mental frames that people are using to construct the reality’ (female participant, research organisation, 3).

‘I think in the last several years about accountability mechanisms that have been put into place by the humanitarian community, in general, to hopefully pick up when people are just doing whatever they feel like it now and not using evidence’ (female participant, INGO, 2).

In some cases, evidence could therefore be used as a tool to reinforce decision-making structures with international agencies at the top. One participant working within an international agency described intervening to change practices led by local actors, using particular notions of internationally recognised evidence to do so:

‘In [a] sub-Saharan African country, when I did a mission there, I saw a local NGO and they did group interventions that I thought were just not aligned with the evidence that we have […] Well we thought this is not good, and we could then… that’s basically the reason for me to advise to ask that NGO to stop doing that or stop their funding’ (male participant, international agency, 6).

Here, the presence of evidence on a particular topic could be used as a way for an international actor changing the practice of a ‘local NGO’. Evidence viewed in this way relied on a perspective of knowledge flowing in one direction, from (international) experts to national or community actors:

‘Then there are cases where national guidelines and evidence or new evidence don’t match, and you can’t change national guidelines in one day’ (female participant, INGO, 1).

‘In the sort of new humanitarian push around localisation and local partnerships, etc, I think, (a) that’s really important, but I also think that if you’re a local actor in, I don’t know, country X, and you did this the way that you’ve always done because you’re a faith-based organisation and you haven’t really been in touch with the global trends on X, Y, and Z, and not normally reading WHO guidance on whatever… [a] proliferation of local actors doing a lot of things [that] may not necessarily all be evidence-based’ (female participant, INGO, 2).

In contrast to these discussions, several participants discussed their perceptions of the power imbalances and inequalities of the humanitarian sector at length. In addition to discussing the constraining nature of a donor-led decision-making and funding cycles within the sector, these discussions centered around how the sector was characterised by power imbalances between (mostly Western) donor governments and communities and countries affected by crises; short-term, firefighting approaches; and a leaning towards feelings of entitlement, impunity and ‘saviourism’. Overall, this lack of accountability to the long-term interests and priorities of populations was described to lead to huge power imbalances and lack of interest and attention to the broader dynamics of the sector and genuine impact on people’s lives:

‘So we are in this exceptional moment and therefore we can sort of recreate, you give yourself power to just sort of you know forget about history and politics and all of that, the rules. There’s an exceptionalism here that you know justifies all of this international dimension, but also allows you to act as if it were free for all zone, free zone’ (male participant, INGO, 12).

Interestingly, for participants who discussed this view, evidence was seen as playing different roles in this dynamic. Some perceived there to be a general lack of interest in evidence in the sector, and that this was reinforced by, and itself reinforced, profound power imbalances and a lack of attention to the standards and appropriateness of humanitarian action:

‘The degree of wacko that can happen when you don’t have robust methodology […] my underlying belief about that is that the same degrees of evidence are necessary whether it’s humanitarian or not. I don’t think you get a free pass […]’

Interviewer: ‘So you’re saying that the humanitarian health field often does get a free pass, do you think?’

‘Yes, I do. I do think they get a free pass. Because I think there’s a lot of biases that go into it and a lot of that just deals with power dynamics of money and the way the world is structured, but just because people are poor or just because they’re quote, unquote, considered to be in a “humanitarian crisis”, doesn’t mean that standard should be less’ (female participant, research organisation, 8).

As this example suggests, some participants implicitly positioned integrating evidence use as a way of making the sector more equitable and accountable to populations affected by crises. A lack of evidence is here described to lead to ‘a free pass’ and lower standards, and was therefore implicitly seen as a way of counteracting such a dynamic.

Evidence could therefore be portrayed as the alternative, at times even binary opposite, of donor-driven priorities, within an overall dynamic that is oriented primarily towards securing funding. In such a view, participants positioned decision-making that prioritised donor interests and fundraising in opposition to evidence-based approaches:

‘How do I think the culture and practices of aid inform the use of evidence and decision-making in response? Well, I just repeat the thing about incentives, which is that it’s a system, it’s an economy. If you think about it as an accountability system – what are the incentives that are in place in that accountability system? What do people get rewarded for? What do they get punished for? What is the currency of reward and punishment? The main currency of reward and punishment in the humanitarian sector is, well, there’s two, I guess, and one of them is access but that’s actually much lower on the totem pole compared to money. It’s money. People don’t get more money for doing stuff that’s evidence-based’ (female participant, research organisation, 3).

‘By and large the organisation wasn’t driven by evidence, it was driven by fundraising priorities, by many other factors that I think you know took priority […] that way of working not only violates do not harm principles, but also results in a lot of moral distress for staff’ (male particiapnt, academia, 10).

In examples such as these, being ‘evidence-based’ and ‘driven by evidence’ were portrayed in contrast to top-down donor-driven decision-making. However, evidence was described to be neglected and almost a casualty of such top-down approaches, rather than being seen as a way of challenging harmful and unequal systems. As the first participant above says, other factors are all ‘lower on the totem pole compared to money’. Evidence could therefore be seen, in these perspectives, as a potential to change the sector for the better, but ultimately thwarted and overruled by funding dynamics.

In contrast, some participants engaging in similar critiques of the sector as a whole felt that donors and international organisations were increasing their focus on evidence. However, that this did not necessarily lead to meaningful and positive change. Evidence was described as becoming a buzzword without meaningful attention to the real-world impact it purports to reflect:

‘Evidence based is a buzzword […] it was about six or seven years ago, you started to have to throw that around and you’d see in the [UN agency] document, you’d see it all around and you just had to have the idea, that what you were doing was evidence-based, and politicians need to be selling that to the public’ (male participant, INGO, 12).

‘Let’s say if you have a major call for proposal for humanitarian programming, there will be three or five different consortia of partners who are bidding for it. They have the incentive to demonstrate that they have the most updated information and they have better understanding of the ground realities, etc. These are typical words that we use. There is an incentive to have a competitive edge over the other, so having evidence is one of the areas to keep that competitive advantage’ (male participant, INGO, 9).

As these narratives suggest, a focus on evidence could also be seen as a tool for generating necessary support and resources within a top-down dynamic, such as to mobilise political support (‘selling that to the public’), and secure funding (‘to keep that competitive advantage’). Evidence being described as a ‘buzzword’ and mention of ‘typical words that we use’, point to discourses around the use of evidence within the sector. For the participants that took this view, an increased focus on generating evidence of programme impact was a tool for securing funding and support, rather than on using it to understand meaningful long-term impact, and was therefore seen as a negative driver for the sector. The types of evidence prioritised within the funding timeframes were felt to be a part of, and indeed further exacerbate, inequalities and harms caused within the sector:

‘It’s more of the output level. We want to treat this many children and everyone is happy if you have treated that many children, but how many have relapsed after the project has ended? Neither donors nor the typical most agencies care’ (male participant, INGO, 9).

In this perspective, a focus on quantifiable outputs, intersecting with short-term funding cycles, could lead to a decreased focus on meaningful impact. A focus on evidence of short-term harm and outputs responding to this were felt to lead to a neglect of long-term interests. One actor at an INGO described this as ‘a triumph of the urgent over the important’, where evidence of short-term, urgent need, could lead to making short-term decisions that led to harm in the long run. Further, the lack of interest in long-term impact was described as leading to a blindness to the potential harm caused to communities by humanitarian assistance. As this actor went on to describe, the harmful impacts of such an approach within the sector would not be captured in a short-term approach to generating evidence:

‘[T]here is no evidence of that, that fuelling of community strife, the building of dependence, the de-responsibilisation of a state ministry of health. None of that is visible within six months and therefore none of that is visible – period, even though you’re in a location for twenty years, especially when you’re rotating people every three months. Because you got people with “six month” lenses on’ (male participant, INGO, 12).

In such a perspective, an increasing focus on evidence could actually become a tool for harm within the sector, and may exacerbate short-term approaches that focused on short-term outputs and did not accurately capture and attend to responsibilities to the long-term interests of communities most affected by crises. Evidence, therefore, could be seen to fuel short-term approaches within the sector that were felt to be at the heart of power imbalances and inefficacies.

In addition, some participants also discussed the fact that processes of evidence production were often top-down and rooted in global and national inequalities:

‘Much of the production of the evidence is still in the Global North as it’s called, I think. That is still happening, that hasn’t changed so much’ (male participant, international agency, 6).

‘Research is driven mainly by Western countries, by the funding from Western countries, but somehow in much more collaborative way with some good and bad, I would say, effect on this. I’ve seen that often Western countries are able to co-opt the elites already […] without necessarily reaching out to those that might have better things to say’ (male participant, international agency, 5).

However while this was acknowledged and discussed by a small number of participants, on the whole inequalities within the production of evidence, and what this meant for what kinds of evidence were valued, were rarely discussed.

### The role of evidence in bottom-up approaches to decision-making

Interestingly, while most participants critiqued the top-down nature of decision-making and there was widespread agreement that the sector did not adequately prioritise accountability to populations most affected by crises, explicit acknowledgement of who this excluded were rare. A small number of participants mentioned how such top-down dynamics within the sector sidelined the views of populations who were central to the action being decided:

‘It’s always the same countries and sometimes with a cook recipe, with limited evidence about what works, and even if they have tools. […] I mean the challenge we are facing in this game is that, how can we really use the existing evidence to influence decisions that are made, often made without the beneficiaries or the country themselves being fully involved?’ (male participant, international agency, 5).

‘…the internal legitimacy and this relates a lot to [what is] quite negatively described the savourism... When you’re saving the world a little bit of, you know […] “don’t pester me with that stuff, we gotta save the world” […] Why doesn’t it engage with communities? Well because that takes time and time is lives and we need to act. It is very much that, I could use the word neo-colonial, [or] paternal, the “we know best”’ (male participant, INGO, 12).

‘I think one danger, in my view, of the push for evidence is that it becomes a top-down approach because collecting evidence in the research way that we talked about, it just requires quite a lot of background and background knowledge and resources, actually. I think that local perspectives tend to be sidelined in those processes’ (male participant, international agency, 6).

In these accounts, engaging with key local populations is presented as a central aspect of humanitarian decision-making, and explicitly mentioned as lacking in such a dynamic. However as in these examples, when exclusions were discussed in this top-down dynamic, these exclusions tended to be discussed in broad terms, such as sidelining ‘communities’, ‘beneficiaries’ and ‘the country themselves’.

When asked about bottom-up approaches to generating evidence that directly sought the views of communities on humanitarian action, participants held differing views on recent changes in the sector. Some felt that the sector had changed significantly and there was now a push to focus more meaningfully on populations most affected by crises. Others felt this process had begun but had not changed a huge amount. One participant, when asked directly about bottom-up mechanisms with information travelling from ‘populations of concern back to decision-makers’, responded that ‘that doesn’t really happen much I’m afraid. That should happen’ (male participant, international agency, 6). Others also held this view: ‘There’s localisation, but it hasn’t really gone down as much as it should’ (female participant, INGO, 1).

Despite acknowledgement that this was important, and some participants viewing the sidelining of crucial communities as a central issue in the sector, few participants tended to focus in-depth on how bottom-up approaches could be strengthened. Discussions on this topic tended to include a general lack of specificity about who this should involve and what it means for appropriate and meaningful decision-making. Below, we examine these dynamics in how two aspects of bottom-up approaches were discussed: evidence on priorities of populations most affected by crises, and the views and expertise of local actors.

### Reflecting the priorities of populations most affected by crises in decision-making

For many participants, bottom-up approaches tended to refer to the inclusion of the views of communities receiving humanitarian assistance. This was widely considered to be important, and some felt there had been a shift in the sector towards valuing the perspectives of populations receiving aid:

‘I do think that [clients] are given more space, I think so. Perhaps it’s my subjective view, but I think there is a space for them to talk and the space to hear their voices and for them to have more representation in in this triangle [of evidence]’ (female participant, academia, 11).

‘I think that culture has completely changed and I alluded to this a little bit about client versus beneficiary. I think that culture has made people change (a) their approach as well as the use of evidence, definitely, and the culture of the client feedback, client responsiveness. Are we doing the right thing? Is this what you need for a start’ (female participant, INGO, 2).

There was a lack of clarity around what kinds of approaches were used for gathering such perspectives, and how these were valued, however some specific approaches were discussed. One approach discussed a number of times where remotely administered surveys were used to elicit feedback on the effectiveness of aid:

‘Similarly on localisation […] we did this aid recipient survey, 5,000 people. We paid [survey provider] who’s quite a reputable mobile phone survey provider’ (female participant, research organisation, 3).

‘You’re familiar with [survey provider]? It only started a few years ago […] they survey, they use mobile technology to survey overtime in a given crisis location people, beneficiaries. And they ask questions like, “Do you feel safer now than you did three months ago?”’ (male participant, INGO, 12).

One participant described her organisation starting from an assessment of needs and including ‘feedback mechanisms’ throughout the process:

‘Very much working and discussing with the communities to what their needs are and approaches before even starting anything so all the way from assessment. In that sense, we have always put in client feedback mechanisms all the way through to monitor our work. That’s something that is, I don’t know, I guess compulsory for us as an organization. We have a checklist of are you doing X, Y, Z? We have to have some mechanism to find out what our beneficiaries, we call them clients […] It’s a choice and we work with them and they’re our client. Just as we’re clients in supermarkets we chose what we want, we’re not forced to take stuff’ (female participant, INGO, 2).

This example pointed to an organisational approach to prioritising the views of populations most affected by crises in decision-making and the action itself; however, it was not clear how this worked in practice. Other methodologies were discussed, although it was also felt that these might not lead to high-quality evidence, or might not be seen as evidence:

‘Maybe with official feedback mechanisms… that would not be evidence-based… they will be more like getting feedback from beneficiaries on how something is working. So there are those systems that have been set up as part of our client responsiveness approaches – where we have periodic client exit interviews, or surveys with a community to get their feedback about services and what can change, but that’s not like done in a research environment. That’s the only, yeah, bottom-up approach that I’m aware of’ (female participant, INGO, 1).

Above, this participant describes systems and approaches in place to capture communities’ perspectives on programming; however, these were seen as ‘[not] evidence-based’ and ‘not done in a research environment’. While seen as important, therefore, this was not considered to be formalised evidence. One participant discussed participatory action research methodologies, but also felt that findings from such methods were not valued highly in the sector:

‘In my field, mental health and psychosocial support, you had very strong activistic streams of psychologists or social workers who would work with populations, very action research type of things. That doesn’t really generate much evidence that counts globally, I must say, and it’s begetting less and less. It’s a pity because they’re really interesting things. I don’t see it happening a lot, that local inventions reach it to the global level’ (male participant, international agency, 6).

On the whole, as this last participant expresses, such methods were described to be rare and were not often discussed by participants. While there was widespread agreement among participants that the perspectives of communities and populations most affected by crises should be included more meaningfully in decision-making, and some felt that this shift had begun, there were few examples or discussions of how this had worked in the past or could work, and such approaches did not tend to be viewed as evidence in discussions of ‘evidence-based’ decision-making.

### Reflecting the expertise of local actors in decision-making

In contrast to a widespread agreement that there should be a greater focus on improving accountability to beneficiaries or communities receiving aid, discussions on how the expertise of local actors could be reflected in decision-making, and the forms of evidence this would entail, were rarer—despite broad recognition that localisation is needed. The most clearly articulated situations participants described of working with actors closely within a national context tended to involve national-level staff, and several participants gave examples of how this was central to humanitarian decision-making, and research and action working well:

‘We involved since the very beginning the Ministry of Public Health. The primary healthcare program coordinator in the ministry, and the epidemiologist, working in the epidemiology departmental, the Ministry of Health, and they were extremely enthusiastic since the very beginning. Even if it took longer to establish the collaboration involving them, and that’s why I guess it took two years and a half to get the whole process through. But it was a very strong backup, then in the working field level, in the design also of the questionnaire, because of course they had the comments and questions based on their knowledge of the context and no one of us had so in-depth as they had. I think this involvement of local health authorities is what has really brought the kind of on-the-spot focus of the whole research. They’ve been interested even in the follow-up of the findings, how can these translate actually into policy change in the way certain health issues are tackled at country level, and how can these be streamlined across all the different organization’ (female participant, INGO, 14).

In this example, the involvement of key specific actors within the national government was seen as crucial to developing a meaningful research tool, and then ensuring impact on policy changes following the research.

When it came to actors who were not national, but acting in more localised ways to specific contexts of decision-making, there tended to be a lack of specificity around who ‘local actors’ were perceived to be, what kinds of expertise such actors may bring, and how this could be reflected in decision-making. In discussions of the importance of understanding local contexts for humanitarian action, participants at times described knowledge about contexts that was seen as essential for decision-making. Local expertise was at times an implicit aspect of understanding local context, but it was unclear the role that was played by such actors, or how information was gathered:

‘If I keep going back to Somalia, one political consideration there is always based on clans […] There, I think there is a lot less evidence because maybe those who are from the country, they understand the clan dynamics better than everyone else’ (male participant, INGO, 9).

Here, the insights of ‘those who are from the country’ are seen here to be essential for a good understanding of the local context; however, these were positioned as outside the formal evidence base. Firstly, through being seen in contrast to evidence (‘there is a lot less evidence’), and secondly, as the role of local experts in such narratives, and the ways in which such information was shared, gathered and valued, were not explicitly made clear.

In addition, when discussing the role of local actors’ expertise and how this could be reflected in decision-making, participants tended to position the importance of this alongside strategies for capacity strengthening. When the expertise of local actors, and the importance of including this expertise was discussed, it tended to be seen in relation to capacity needs or the importance of increasing training and expertise in evidence-gathering, in order to fit more easily into internationally recognised methods of generating and collating evidence in the sector:

‘I think training of staff so that they can generate evidence as they work and integrate evidence in their routine service delivery. I feel it’s a missed opportunity where we could generate evidence, good evidence in in a reasonable manner’ (female participant, INGO, 1).

‘I think there have been some brilliant things that have come through, but they’re one in a million and then there must be many others that we just don’t know of or even if they’re just ideas, that we just haven’t been able to capture because A, we’re not exposed to them, B, that person doesn’t think this is something interesting that might be evidence because that’s not what they’ve ever been taught. They’ve not been brought up in the scientific evidence framework […] On a basic level you’re a really busy nurse and you’re just working at this health post and you’re seeing 300 patients or kids a day. You just notice this is a better way of doing something or that approach means that mothers come back for vaccination every time or they all come back for antenatal care. Would we know that? No, she does […] Does she write it down, does she publish it, does she know the [unclear]? No. Maybe it only works for her because she’s a really nice person but no one has… She’s not taught to say, “Look at my success rate and et cetera.” I think plenty of things that possibly get lost’ (female participant, INGO, 2).

As these examples show, at times, questions around feedback mechanisms from the bottom-up became centered around issues of capacity building and training of local actors, rather than centering the perspectives of communities themselves. Here this participant from an INGO views the expertise and knowledge of a nurse as highly valuable, yet needing training to better share this knowledge (‘they’ve never been taught’). Such expertise in these descriptions was seen as valuable, but dominant perspectives viewed this as needing some training to adapt or ‘filter’ local expertise to fit top-down approaches:

Interviewer: ‘I’m wondering if you can think of any other kind of bottom-up mechanisms through which evidence or information on the implementation of humanitarian interventions can travel back from populations of concern to decision-makers?’

‘ […] We’ve started a project on strengthening field-level learning, and as part of that, we’ve developed a resource pack using action-learning methodology […] This piece of work is looking at action learning as a set of approaches to help address that gap I was mentioning between tacit knowledge and formal knowledge, so we’d be using these mechanisms both for field staff to better engage in more structured learning processes for themselves at field level, using what they have with them and in front of them, and using reflective exercises on their own experience. Then also, we have a mechanism for filtering that experience in a more structured way so that it can be shared and used more easily and therefore, valued better’ (female participant, research organisation, 3).

Discussions around the expertise of local actors interestingly tended to be framed in a conceptualisation of ‘lack’ and of what increased training or support would be needed to respond to this lack. As part of the expertise being documented and shared, it made sense for top-down frameworks to be used, with less attention to how the structures could shift and change to accommodate a range of forms of expertise meaningfully. Further, in discussions about the expertise of local actors, there was a sense that knowledge and information about the context flowed upwards, while expertise and capacity for generating and documenting evidence flowed downwards.

There were some exceptions, however, and those who discussed this explicitly felt very strongly about the importance of meaningfully gathering and reflecting both the views of communities most affected by crises and the expertise of local actors. One participant discussed at length how ‘evidence-based’ approaches could only be considered as such if formalised evidence was combined with the views of populations using services and clinical expertise of local actors, all crucial components of being ‘evidence-based’:

‘[F]or instance, in Ukraine […] so we were teaching first psychological aid and this is really evidence-based guide for field workers how to approach what to do, what to not to do. But still, this is this one pillar. So where is the clinical expertise and where is the Ukrainian clients’ voice? […] I could say that yeah we are delivering evidence-based interventions to them because all these three components are combined. Because […] clients and patients and beneficiaries, or whoever users of the services, are acting as a focal points, they are involved in the processes.

[…]

Otherwise you are somewhere in the clouds and you know it is the best evidence in the field and you are really giving that to your co-workers, but also are sensitive and open enough to to hear from them and recognise what is happening. Then you are really enriching your... whatever it’s called.. capacity building, because you are bringing evidence [and] your experience and coupling that with field workers’ experiences and voices of the clients’ (female participant, academia+, 11).

This participant discusses the centrality of both local actors and populations receiving, or using, humanitarian assistance, at all stages of the process of humanitarian action. Here she locates this as central to the concept of what evidence is, as one of its core ‘components’, and then also describes an example of how this worked in practice.

## Discussion

In this paper, we explore the use of evidence in humanitarian decision-making within the context of a localisation agenda. Specifically, we examine the kinds of evidence humanitarian health actors draw on when making decisions, and how the priorities of populations most affected by crises and the expertise of local actors are reflected in such decision-making. We contribute to new understandings of humanitarian narratives at the intersection of ‘evidence’ and ‘localisation’. Our findings draw attention to the ongoing discursive relevance of being ‘evidence-based’ within the humanitarian sector, including how this narrative may perpetuate multiple forms of top-down decision-making structures and diminish the voices and perspectives of populations affected by crises and local actors. We suggest that evidence and localisation appear in humanitarian actors’ narratives as mutually exclusive, however there are missed opportunities for the humanitarian sector to strengthen the implementation of each concept by finding synergies between them.

Our study finds clear hierarchies of what counts as good evidence, including a reliance on quantitative approaches and a leaning towards RCTs as the strongest form of evidence. Aligning with literature, participants did recognise the importance of intuition and experience as evidence (Knox Clarke and Campbell 2020), but tended to label these as less reliable and lacking in rigour. Knowledge of the context was not necessarily seen as evidence by participants. We did not see consistent approaches to how the perspectives of populations most affected by crises were gathered, and this also tended to not be seen as robust evidence. While a small number of participants did position such perspectives and the expertise of local actors as important forms of evidence, they saw themselves in contrast to the rest of the sector. Discussions of evidence were frequently underpinned by notions that certain forms of evidence were inherently objective or reflected scientific ‘truth’, in contrast to growing critiques of such discourses and recognition that evidence is political (Colombo and Checchi 2018; Knox Clarke and Campbell 2020). The implications of reliance on objectivity of evidence are significant, and help to explain why humanitarian actors may be more suspicious of knowledge that they do not see as ‘evidence’, including local expertise. Our paper contributes to growing narratives that knowledge production is political—which is not just relevant to the humanitarian sector but public health more broadly.

The emphasis on objectivity also contextualises comments about the need to build the ‘capacity’ of local actors rather than taking their knowledge at face value, suggesting the idea of objective truth (which certain actors communicate to others) persists. The persistence of the narrative that local actors need capacity is concerning—this is a longstanding critique of humanitarian action (Kothari 2005; Smilie 2001; Taela 2023; Usen 2019). The focus on building capacity sits at odds with the localisation agenda but also explains to some degree why localisation has had such little traction (ALNAP 2022; Metcalfe-Hough et al. 2022; Roepstorff 2020). The focus on the ‘lack’ of local actors may be a result of contrasting seemingly objective research with the knowledge of local actors, including comparisons between technical guidance (perceived as correct and representing the best way to implement) and local practice (perceived as incorrect and clashing with technical guidance).

Our findings reveal a disconnect and tension between evidence and localisation; however, this clash has not been explored within the literature on these concepts. We argue this potential mismatch does a disservice to the implementation of both agendas and propose that instead, finding synergies between these concepts would strengthen both. We suggest evidence needs to be repositioned as not bias-free but in fact political and laden with the interests and motivations of humanitarian actors including donors (Paulus et al. 2023). More work should be done with humanitarian actors to challenge notions of evidence objectivity, including greater consideration of how positionality influences research (Lokot 2022). We suggest humanitarian actors need to better reflect on biases in their decision-making (Comes 2016) and broaden what they consider to be evidence in light of critiques of notions of causality, attribution and impact (Eyben 2013). Rather than focusing on ‘what works’ in the context of efficiency and impact, perhaps evidence itself should shift to explore how communities understand their challenges, how locally-driven solutions might support change, and create more meaningful impact (Eyben 2013; Kothari 2005; Smilie 2001). We find that there is a need for more robust tools and methodologies to capture the views and perspectives of both local experts and populations most affected by crises, and for these tools and methodologies to be shaped according to how such perspectives may be most meaningfully captured—rather than a focus on how they may be made to fit current, widely used tools.

We also suggest humanitarian actors would benefit from more critically considering how the structures perpetuating top-down decisions in the sector need to shift (Lokot and Wake 2021; Mulder 2023). This may include pushing back on attempts by donors to shape evidence-generation agendas and adopting approaches to generating evidence that better centre the voices of populations affected by crises and local actors (Mosse 2007). Lastly, the call for humanitarian actors to take time to listen to populations affected by crises is not a new one (Anderson et al. 2012; Lokot 2019), but bears repeating in light of humanitarian actor narratives that seem to devalue local and lived experience. Emphasising the importance of listening also reaffirms the need for a shift from upwards to downwards accountability (Chynoweth 2015).

## Data Availability

The participants of this study did not give written consent for their transcripts to be shared in a public repository, so due to the sensitive nature of the research supporting data is not available.

## Abbreviations

ALNAP: Active Learning Network for Accountability and Performance
DFID: Department for International Development
RCT: Randomised-controlled trial
RECAP: Research capacity strengthening and knowledge generation to support preparedness and response to humanitarian crises and epidemics
NGO: Non-governmental organisation
UNFPA: United Nations Population Fund
WHO: World Health Organisation
